# Component Characterization, In Vitro Activities and Molecular Mechanism of *Cydonia oblonga* Mill. against Diabetic

**DOI:** 10.3390/ph15121566

**Published:** 2022-12-15

**Authors:** Bingqing Chi, Xilong Liang, Lihua Wang, Yifei Bian, Meng Zhang, Zhixin Tang, Danyang Wang, Zhenhua Tian

**Affiliations:** 1 College of Pharmacy, Shandong University of Traditional Chinese Medicine, Jinan 250355, China; 2 Department of Biostatistics, School of Public Health, Yale University, New Haven, CT 06489, USA; 3 Innovation Institute of Chinese Medicine and Pharmacy, Shandong University of Traditional Chinese Medicine, Jinan 250355, China; 4 Experimental Center, Shandong University of Traditional Chinese Medicine, Jinan 250355, China

**Keywords:** *Cydonia oblonga* Mill., component characterization, diabetic, antioxidant activity, α-glucosidase inhibitory activity

## Abstract

*Cydonia Oblonga* Mill. is widely distributed in Turkey, Uzbekistan and China and commonly used by the food industry to produce jam, jelly and candies. The aim of this study was to investigate the in vitro antidiabetic activity and anti-diabetic mechanism of *Cydonia Oblonga* Mill. fruit (COMF). The chemical compositions were further characterized in COMF by UPLC-Q-Orbitrap/MS and 65 components including 22 flavonoids, 16 organic acids, 11 polyphenols, 5 amino acids, 3 pentacyclic triterpenoids and 8 other compounds were identified. The antioxidant activity by DPPH scavenging method and α-glucosidase inhibitory activity were tested. Furthermore, we detected the effects of COMF extract on the proliferation activity of HUVECs, cell viability of HUVECs under H_2_O_2_-induced oxidative stress, and NO production. Then, molecular docking activity and α-glucosidase inhibitory activity of seven key flavonoid components selected by bioinformatics analysis and literature in the COMF were studied. Among them, quercetin showed potent inhibitory activity, kaempferol, isorhamnetin, luteolin and apigenin demonstrated moderate inhibitory activity, while rutin and epicatechin exhibited poor inhibitory activity. Subsequently, the effects of quercetin, kaempferol, isorhamnetin, leteolin and apigenin on the gene expression levels of AKT1, IL-6 and VEGFA were verified by real-time fluorescence quantification (RT-qPCR). Molecular biology result showed that different active ingredients can significantly recover the levels of AKT1, IL-6 and VEGFA in HUVECs injured by high glucose.

## 1. Introduction

Diabetes mellitus (DM) is a group of metabolic diseases characterized by chronic elevated blood glucose. According to the International Diabetes Federation (IDF), 537 million (10.5 %) adults aged 20–79 have DM, and more than 1.2 million children and adolescents have type 1 diabetes mellitus (T1DM). DM caused 6.7 million deaths in 2021 [1]. DM has become a major public health problem that threatens the health of citizens worldwide. DM is caused by a relative or absolute shortage of insulin. At present, the pathogenesis of DM is mainly believed to be insulin resistance and impaired islet β cell function [2]. Insulin could exert its hypoglycemic effect by regulating the PI3K/AKT signaling pathway and APS signaling pathway, and then affect the rate of glucose uptake by cells [3]. Excess glucose in the circulatory system will damage the PI3K-AKT signal transduction pathway in tissues, resulting in abnormal lipid deposition, glucose transport, glycogen synthesis and gluconeogenesis in peripheral insulin-sensitive tissues (liver and skeletal muscle), and leading to islet β-cell dysfunction and abnormal insulin secretion [4,5]. Sulfonylureas, diphenyldiamines and thiazolidinediones are commonly used in clinical treatment of DM [6], but these drugs have limited selectivity and have toxic side effects on kidney and liver. In recent years, the research on anti-diabetes of natural medicine and ethnic medicine has attracted much attention. According to previous study, natural products against diabetes mainly include alkaloids, flavonoids and saponins [7]. Natural plants stand out among novel drugs for the treatment of diabetes due to their low toxicity and multi-target synergistic effects. Based on the characterization of plant components, further study on the pleiotropic activities of anti-diabetic plants provides a research idea for screening natural anti-diabetic plants [8].

*Cydonia oblonga* Mill. (COM) is a plant of the genus Cydonia in the Rosaceae family [9]. COM originated in the Caucasus Mountains between Persia and Turkmenistan [10]. COM grows well in fertile loam with abundant sand grains, and also in saline soil. COM has the functions of food, medicine and health care in its folk use. Its fruits, seeds, leaves, branches and roots can be used as medicine. COM, as a traditional plant medicine, is used to prevent or treat cancer, diabetes, hepatitis, ulcers, respiratory and urinary tract infections and other diseases. Fresh *Cydonia oblonga* Mill. fruit (COMF) has an unignorable astringency and acidity. The mature fruit of selected COM varieties, however, has a wonderful sweet and sour taste. Today, COMF products such as jams, jellies, cakes and wine are popular in many countries [11]. As a characteristic herbal medicine, COMF contains rich nutritional compounds, such as phenolic acids, flavonoids, lignin and other active ingredients. Phenolic compounds mainly consist of caffeic acid and its derivatives, coumaric acids and other ingredients [12]. Flavonoids include flavanols, flavonoids and flavonoid glycosides, isoflavones and so on [13]. COMF possesses antioxidant, antibacterial and anti-inflammatory properties [14,15]. COMF flavonoids have various biological activities, such as hypotensive, hypolipidemic, anti-inflammatory and antibacterial effects.

In the past decade, COM has been reported to treat diabetes or hyperlipidemia. COM leaves may play a role in hypolipidaemic and hepatoprotective by improving the antioxidant capacity and lipoprotein metabolism of liver to inhibit lipogenesis [16]. Dan Tang studied the effect of COM on glucose metabolism, and its mechanism is related to the activation of PI3K-AKT insulin signaling pathway [6]. However, reports on COMF treatment of hyperlipidemia or diabetes generally only involve the mechanism of action of total extract, and the potential active ingredients have not been thoroughly studied.

Therefore, this study first determined the in vitro activities of COMF, including the DPPH (1,1-diphenyl-2-picryl-hydrazyl radical) radical scavenging activity, antioxidant stress activity on H_2_O_2_-induced HUVECs cells and α-glucosidase inhibition activity. Moreover, the α-glucosidase inhibition activity showed its inhibition rate was positively correlated with its concentration and the rate was up to 95.99%, which encouraged to further explore its material basis. Then, this study established a UPLC-MS/MS method for analyzing active components of COMF and deduced its fragmentation pattern. Based on component characterization and bioinformatics data mining, seven active ingredients (flavonoids) were selected for computer prediction and in vitro validation of α-glucosidase inhibitory activity. As for the molecular mechanism of anti-diabetes, five compounds with better activity in vitro were selected to explore their effects on the AKT1, IL-6 and VEGFA mRNA expression, indicating that their anti-diabetes mechanism was related to PI3K-AKT signaling pathway.

## 2. Results

### 2.1. Identification of COMF Components

**Figure 1:**
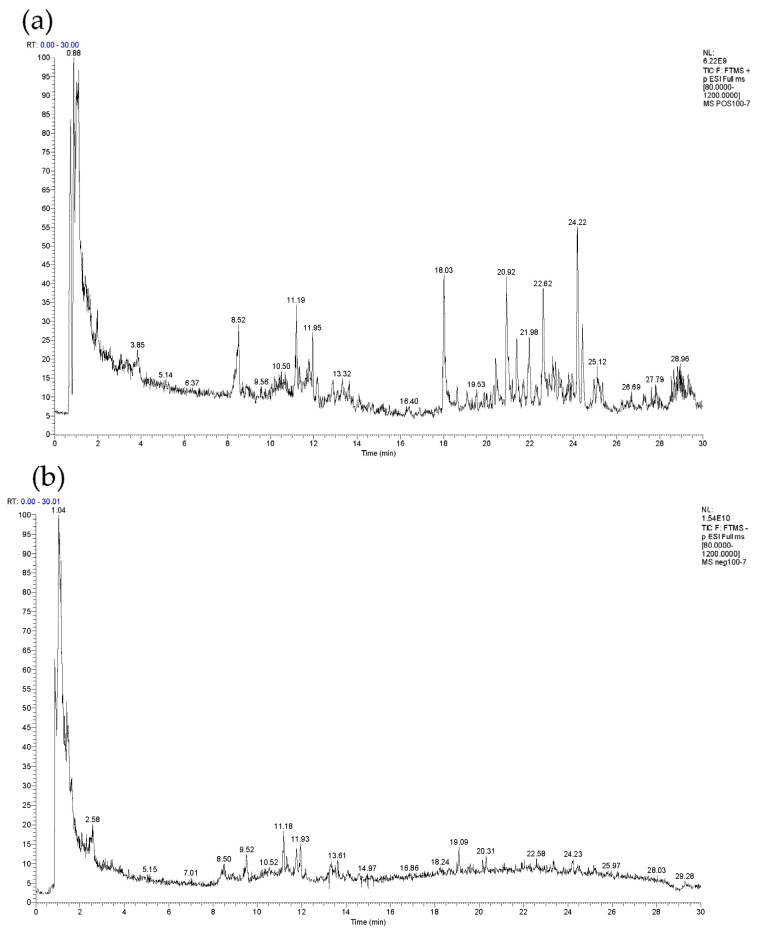
TIC spectrum of COMF extract: (**a**) ESI (+) -TIC scanning mass spectrometry; (**b**) ESI (−) -TIC scanning mass spectrometry.

In order to characterize the active components of COMF, the extract of COMF was analyzed by UPLC-MS/MS. The total ion chromatogram (TIC) of COMF extract was shown in Figure 1. The LC-MS data were imported into Xcalibur software (version 3.0.63), and the chemical formula was deduced according to the high-resolution mass spectrometry information. The prediction deviation is within ±10 ppm. The possible compounds in COMF were deduced by referring to references and online databases and combining with the fragmentation pattern of 20 standard substances. Finally, 65 compounds identified in COMF were listed in Table 1 including retention time, precursor ions, detected and calculated m/z value, error (ppm), molecular formula, MS fragment ions and compound name.

**Figure 2:**
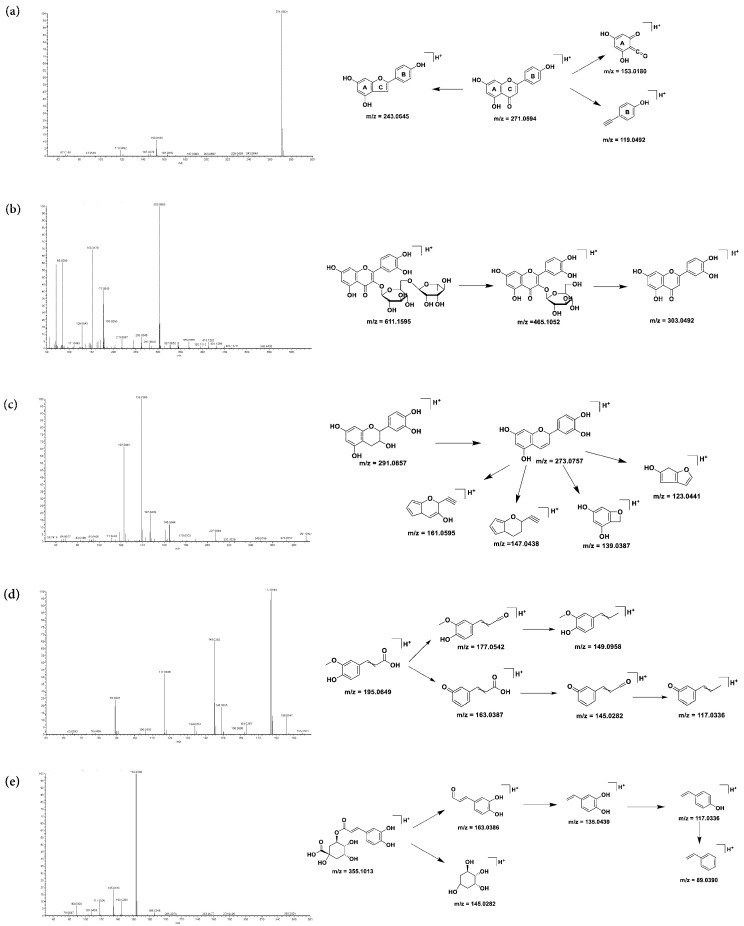
MS^2^ spectrometry and fragmentation pattern of compounds in positive ion mode: (**a**) apigenin (flavonoids); (**b**) rutin (flavonoid glycoside); (**c**) catechin (flavane-3-alcohol); (**d**) chlorogenic acid (caffeylquinic acid); (**e**) ferulic acid (cinnamic acid derivative).

According to the analytical results, the compounds included 22 flavonoids, 16 organic acids, 11 polyphenols, 5 amino acids, 3 pentacyclic triterpenoids and 8 other compounds. Flavonoid, organic acid and polyphenol were the main active compounds of COMF. Different types of compounds have different fragmentation pattern (Figure 2). Unknown compounds in COMF can be identified by analyzing the cracking patterns of standard compounds for each type of compound.

#### 2.1.1. Fragmentation Pattern of Flavonoid Compounds

Flavonoids have the basic structure of C6-C3-C6 and form rings A, C and B, respectively. The [M + H]^+^ of free flavonoids is relatively stable, so the molecular ion peak is generally the base peak. In addition, there are [M + H − CO]^+^, fragment ions of A ring and fragment ions of B ring formed after fracture of C ring. The fragment ions of apigenin standard accord with the cracking law of free flavonoids completely. The base peak is 271.0594 [M + H]^+^, the fragment ion is 243.0645 [M + H − 28]+ after losing A molecule of CO, and 153.0180 (A-ring fragment ion) and 119.0492 (B-ring fragment ion) are formed after cleavage of ring A. The cleavage law of peaks 46 and 50 were similar to apigenin, with [M + H − CO]^+^, A-ring fragment and B-ring fragment, which were identified as naringin and luteolin based on standard substance and literature.

Compounds **59** and **60** are isomeric and have relatively stable molecular ion peaks. [M + H]^+^ loses one molecule of CH_3_ and one molecule of CO successively, and the fragment ions tend to be stable. Therefore, 59 and 60 can be judged to be flavonoids with methoxyl group at the 6th or 8th position of ring A. Compared with the literature, 59 and 60 were speculated to be sakuranetin and isosakuranetin.

Rutin (Compound 33), as a typical flavonoid glycoside, tends to lose rhamnose [M + H − 146]^+^ and then break glucose [M + H − 146 − 162]^+^ in the process of ionization. At this time, the flavonoid aglycones were relatively stable and followed the flavonoid cracking rule. Compounds **35, 36, 37, 39, 42, 44** and **45** had similar pyrolysis patterns with rutin, and were identified as hyperin, isoquercitrin, luteolin, kahenol rutin glycoside, astragalin, hesperidin and quercitrin, respectively.

3-flavanols can be used as precursors of tannins in plants, which are usually in the form of molecular polymerization. In this study, four flavane-3-alcohols were identified. Taking the standard catechin as an example, the cleavage fragments of flavane-3-alcohol parent nucleus were analyzed as 273 [M + H − H_2_O]^+^, 161 [M + H − C_5_H_5_O_4_]^+^, 147 [M + H − C_5_H_4_O_5_]^+^, 139 [M + H − C_8_H_7_O_3_]^+^, 123 [M + H − C_8_H_7_O_4_]^+^. Compounds **15**, **18** and **22** were identified as procyanidin B1, procyanidin B2 and epicatechin by comparison with cate-chin in terms of retention time, parent and product ions.

#### 2.1.2. Organic Acid and Polyphenol Compounds

The main fracture modes of caffeylquinic acid compounds are ester bond fracture and oxy bond fracture, and loss of neutral molecules H_2_O and CO_2_. The molecular ion peak of compound **21** was 355.1013 [M + H]^+^. A fragment ion with m/z 145.0282 was obtained by the loss of caffeyl and ester groups. Meanwhile, the coffee acyl fragment ion (m/z 163.0386) gradually lost a molecule of CO (m/z 135.0439), a molecule of H_2_O (m/z 117.0336) and a molecule of H_2_O to obtain m/z 89.0390 fragment ion. Compound **21** was identified as chlorogenic acid according to the standard substance and references. Compounds **16** and **27**, isomers of chlorogenic acid, were identified as neochlorogenic acid and cryptochlorogenic acid accordingly.

Isochlorogenic acids are compounds of diccoffeylquinic acid, which has similar fragmentation pattern to chlorogenic acid. Compounds **41**, **43**, and **47** all lost a molecule of H_2_O to obtain the fragment ion m/z 499.1225, and the caffeyl group was separated from the quinic acid skeleton to obtain the fragment m/z 163.0386. The caffeyl fragment loses CO and H_2_O. Compounds **41**, **43** and **47** were identified as isochlorogenic acid B, A and C by comparison with the standard substance.

The characteristic fragments of cinnamic acid are [M + H − H_2_O]^+^ and [M + H − H_2_O − CO]^+^. Compounds **31**, **32** and **40** have similar fragment ions. They were identified as 2-hydroxycinnamic acid, 4-hydroxycinnamic acid and cinnamic acid. For ferulic acid, m/z 177.0542 is obtained by losing a molecule of H_2_O, and m/z 149.0958 is obtained by losing another molecule of CO. In addition, ferulic acid molecules may lose CH_2_ and H_2_O fragments first to obtain m/z 163.0387, and then lose H_2_O and CO to obtain m/z 145.0282 and 117.0336.

#### 2.1.3. Other Compounds

The structure of pentacyclic triterpenes was stable under different cracking voltages, and the fragment ions lost were mostly -CH_3_, -OH, -COOH and so on. Organic acids characteristically lose fragments of -CO_2_ (m/z 44), such as the succinic acid fragment ions m/z 117 to m/z 73, which lose a molecule of CO_2_.

### 2.2. In Vitro Activity Assay

#### 2.2.1. DPPH Free Radical Scavenging Ability of COMF Extract

**Figure 3:**
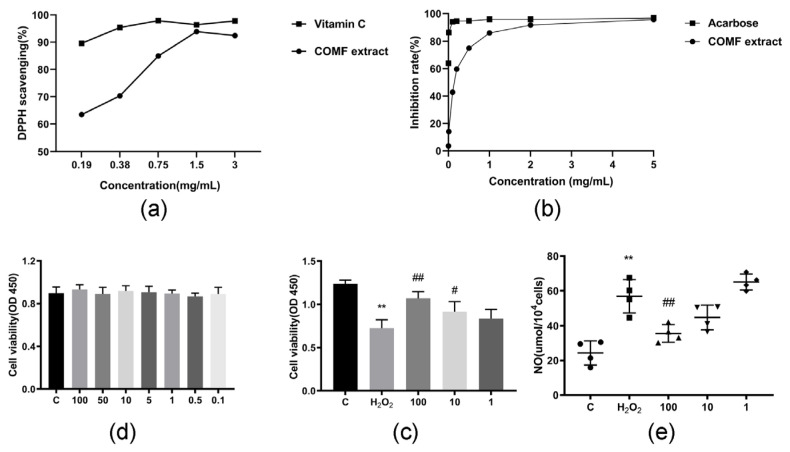
COMF extract in vitro activity assay. (**a**) DPPH clearance rate of COMF extract and ascorbic acid (positive control) at different concentrations (%); (**b**) inhibitory rate of acarbose (positive control) and COMF extract on α-glucosidase (%); (**c**) cell activity as measured by CCK-8 kit. HUVECs were not damaged by hydrogen peroxide; (**d**) cell activity as measured by CCK-8 kit. HUVECs are hydrogen peroxide-stimulated cells; (**e**) NO content in HUVECs. (Using NO assay kit). * represents a significant difference from group C (blank group), ** indicates *p* < 0.01. # represents a significant difference compared with H_2_O_2_ group (model group), # indicates *p* < 0.05 and ## indicates *p* < 0.01.

DPPH is a stable free radical with simple molecular structure and has been used to evaluate the antioxidant activity of natural products in vitro. As shown in Figure 3a, the antioxidant capacity of the positive control ascorbic acid and COMF extracts increased in a dose-dependent manner between the concentration range of 0.19–3.00 mg/mL. Finally, it is leveled off. The IC_50_ of ascorbic acid and COMF extracts were 0.004 and 0.108 mg/mL.

#### 2.2.2. α-Glucosidase Inhibitory Potency

The α-glucosidase inhibitor acarbose has strong inhibitory effect on α-glucosidase in clinical application. Therefore, a series of concentrations of acarbose were selected as positive controls to determine the α-glucosidase inhibitory activity of COMF extract and active monomer at the same concentration. As can be seen from Figure 3b, the inhibition rate of COMF extract was positively correlated with its concentration. The inhibition rate of the extract was up to 95.99%.

#### 2.2.3. Effect of COMF Extracts on HUVECs Proliferation

Firstly, the effect of COMF extract on the proliferation of HUVECs was tested. As shown in Figure 3c, COMF (100, 50, 10, 5, 1, 0.5 and 0.1 μg/mL) for 24 h showed no significant toxicity to HUVECs (*p* > 0.05), indicating that there is no cytotoxicity observed for the extracts of COMF in the tested range.

#### 2.2.4. Cytoprotective Effect of Extracts in H_2_O_2_-Induced Oxidative Stress

HUVECs were pretreated with COMF extract (100, 10 and 1 μg/mL) for 24 h. As expected in Figure 3d, survival in the model group was significantly different from that in the control group (*p* < 0.001). In addition, 10 and 100 μg/mL COMF showed a protective effect (*p* < 0.05 and *P* < 0.001), and only at 1 μg/mL COMF showed no significant difference (*p* > 0.05) from the model group.

#### 2.2.5. The Extracts Effects on NO Production

HUVECs were pretreated with H_2_O_2_ and co-cultured with COMF extract. The sup-pression effect of COMF on NO was detected by NO Content Detection Kit. The results were shown in Figure 3e, which demonstrated that 100 μg/mL COMF extract had strong inhibitory effect (*p* < 0.001). However, 10 and 1 μg/mL COMF extract could not significantly inhibit NO production in stressed HUVECs (*p* > 0.05).

### 2.3. Selection of Active Monomers

The outputs of Cytoscape include the average shortest path length (ASPL), betweenness centrality (BC), closeness centrality (CC) and degree relevance judgment factors (Table 2). Based on the results and the literature, quercetin, kaempferol, isorhamnetin, leteolin, apigenin, rutin and epicatechin were finally selected as the active ligand for further study.

### 2.4. In Vitro Activity Assay Result and Validation of Molecular Mechanisms

#### 2.4.1. α-Glucosidase Inhibitory Potency

**Figure 4:**
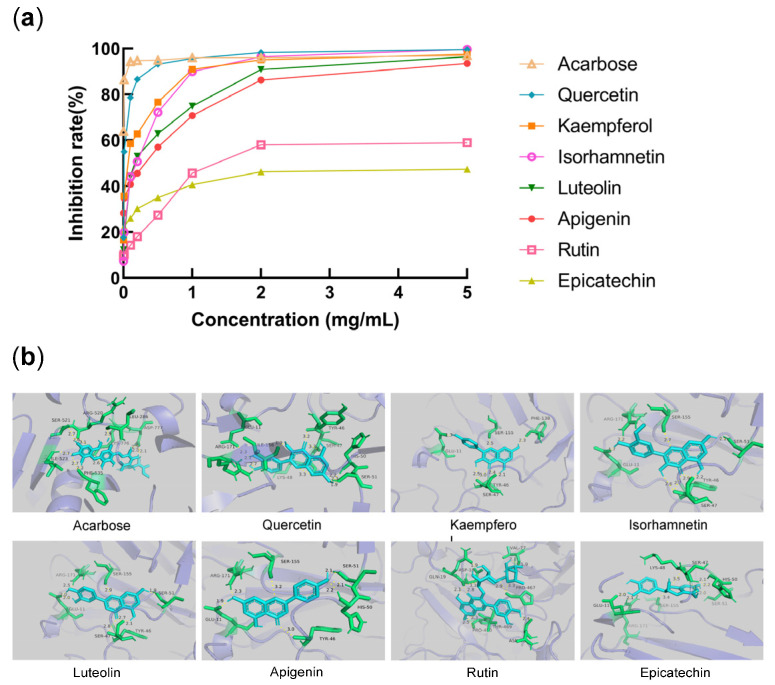
The date of flavonoids with α-glucosidase inhibition activity and molecular docking: (**a**) acarbose (positive control) and active constituents on α-glucosidase inhibition activity; (**b**) molecular docking analysis of acarbose (positive control) and active constituents.

The α-glucosidase inhibitor acarbose has strong inhibitory effect on α-glucosidase in clinical application. Therefore, a series of concentrations of acarbose were selected as a positive control to determine the α-glucosidase inhibitory activity of COMF extract and active monomer at the same concentration. As can be seen from Figure 4a, the inhibition rate of COMF extract was positively correlated with its concentration. The inhibition rate of the extract was up to 95.99%.

Table 3 and Figure 4b shown the IC_50_ value of the studied plant active monomer on the inhibition rate of α-glucosidase. The results revealed that the IC_50_ value of quercetin was 0.0090 mg/mL, and the inhibitory effect was remarkable. The IC_50_ values of kaempferol, isorhamnetin, luteolin and apigenin were in the range of 0.0646–0.2508 mg/mL, and they also had good effects. The IC_50_ values of rutin and epicatechin were 1.4790 mg/mL and >5.0000 mg/mL, respectively, and the inhibitory effect was poor. The inhibitory effect of the seven flavonoids on α-glucosidase was consistent with the result of molecular docking.

#### 2.4.2. Effect of COMF Extracts and Active Compounds on HUVECs Proliferation

**Figure 5:**
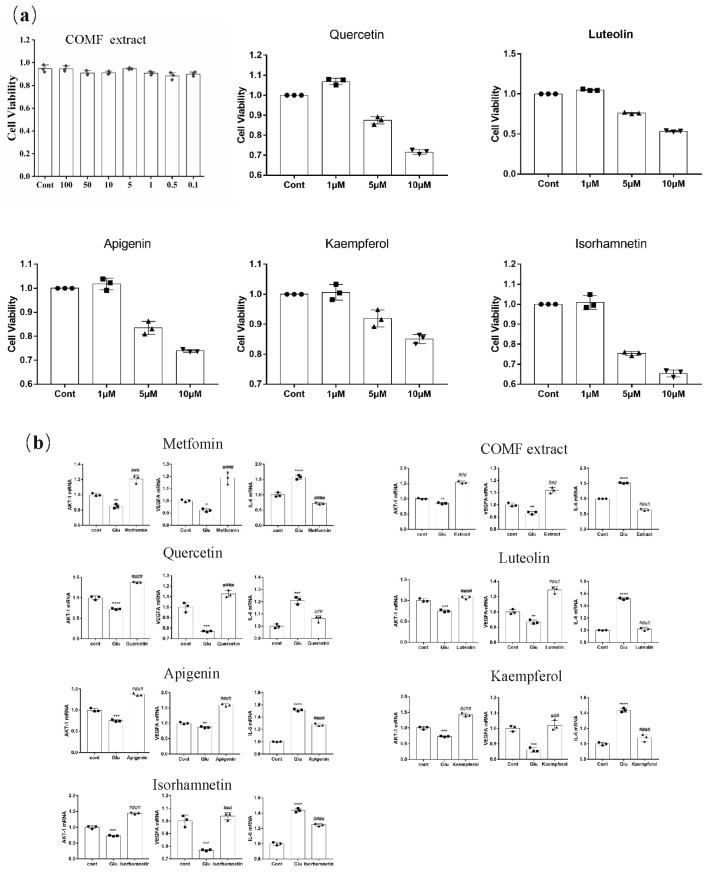
mRNA expression of extracts and active ingredients. (**a**) Cell activity as measured by CCK8 kit. (**b**) Expression of AKT1, VEGFA, IL-6 mRNA. * represents a significant difference from group Cont, * indicates *p* < 0.05, ** indicates *p* < 0.01, *** indicates *p* < 0.001 and **** indicates *p* < 0.0001. # represents a significant difference compared with group Glu, ### indicates *p* < 0.001 and #### indicates *p* < 0.0001.

The effect of COMF extract on the proliferation of HUVECs was tested. As shown in Figure 5a, COMF (100, 50, 10, 5.0, 1.0, 0.5 and 0.1 μg/mL) and active compounds in 1.0 μM for 24 h showed no significant toxicity to HUVECs (*p* > 0.05), indicating that there is no cytotoxicity observed for the extracts of COMF in the tested range. The active ingredients of 5.0 and 10 μM affected the cells to different degrees, so it was decided to use the 1.0 μM concentration for the subsequent experiments.

#### 2.4.3. Expression of AKT1, IL-6 and VEGFA mRNA

Compared with the blank group, the expression of IL-6 mRNA in the model group was significantly up-regulated (*p* < 0.01). Compared with the model group, the IL-6 mRNA expression levels of quercetin, kaempferol, isorhamnetin, luteolin and apigenin group were significantly down-regulated. Luteolin group was the most significant down-regulation. Compared with the blank group, the mRNA expression levels of AKT1 and VEGFA in the model group were significantly down-regulated (*p* < 0.05). Compared with the model group, the expression levels of AKT1 and VEGFA mRNA of the five active compounds were significantly up-regulated (Figure 5b).

## 3. Materials and Methods

### 3.1. Sample Preparation

*Cydonia oblonga* Mill. fruits were collected in January 2020 in Xinjiang, China, and identified by Professor Hongyan Liu (Shandong University of Traditional Chinese Medicine, Jinan, China). The voucher specimen (No. 2020-COMF) was stored in the department of Pharmaceutical Chemistry of Shandong University of Traditional Chinese Medicine.

A total of 10 g of fruit was ultrasonically twice with 100 mL methanol for 30 min, and the solution was combined and filtered. The resulting liquid was concentrated in a rotary evaporator and the yield was 18.31 % (*w*/*w*). The extract was dissolved in methanol and filtered through a 0.22 μm microporous filter.

### 3.2. UPLC-MS/MS Analysis

The extract was analyzed by Thermo Fisher UltiMate 3000 UPLC (Thermo Fisher Scientific, Waltham, MA, USA). A Waters ACQUITY UPLC HSS T3 (2.1 × 100 mm, 1.8 μm) column was used with mobile phases of 0.05% formic acid aqueous solution (A)—0.05% formic acid acetonitrile (B). Gradient elution (0–5 min, 0% B; 5–15 min, 0–33% B; 15–20 min, 33–63% B; 20–30 min, 63–2% B). Column temperature was 30 °C. Flow rate was 0.3 mL/min, and injection volume was 5 μL.

The mass spectrometry was performed using Thermo Fisher Q-Exactive Orbitrap MS system (Thermo Fisher, MA, USA). ESI ion source was adopted positive and negative ion modes. For the positive and negative ESI analysis, the parameters were as follows: capillary voltage at 3500 V, temperature at 350 °C, sheath gas at 45.0 L/min, auxiliary gas at 10.0 L/min, fragmentation voltage at 60 V, collision energy at 20.0, 40.0 and 60.0 eV. Quality scan range was 80–1200 Da.

### 3.3. In Vitro Activity Assay

#### 3.3.1. Determination of DPPH Clearance Rate

The scavenging capacity of DPPH was determined by Costa et al. [35]. A total of 100 μL of the extracts with different concentrations (0.19, 0.38, 0.75, 1.5 mg/mL) and 100 μL 0.1 mM DPPD solution (dissolved in absolute ethanol) were incubated at 37 °C for 30 min away from light, and the absorbance at 417 nm was measured with a microplate reader. Ascorbic acid was used as positive control. DPPH clearance rate (%) is calculated by Formula (1), as follows:

Clearance rate (%) = (A_1_ − A_2_)/A_3_ × 100%, (1)

where A_1_ is the absorbance of sample extract and DPPH; A_2_ represents the absorbance of sample extract and anhydrous ethanol solution; A_3_ is the absorbance of DPPH and anhydrous ethanol solution; IC_50_ is the sample concentration of 50% DPPH scavenging activity.

#### 3.3.2. Determination of α-Glucosidase Inhibition Rate

The α-glucosidase inhibitory activity was measured by referring to the method of Cai et al. [36]. 20 μL COMF extract (0.001, 0.01, 0.1, 0.2, 0.5, 1.0, 2.0, 5.0 mg/mL) and 20 μL 0.4 U/mL α-glucosidase were mixed in 120 μL 0.5 M potassium phosphate-buffered solution (PBS, pH 7.4). Then, 50 μL 3 mM P-NPG solution was added and incubated at 37 °C for 1 h. Finally, 50 μL 0.67 M sodium carbonate solution was added to terminate the reaction. Absorbance was measured at 405 nm (A). The positive control was acarbose. The inhibition rate (%) was calculated according to Formula (2):

Inhibitory rate (%) = [1 − (A_2_ − A_3_)/A_1_] × 100%, (2)

where A_1_ represents the absorbance of buffer and enzyme system; A_2_ represents the absorbance of sample extract and enzyme system; A_3_ represents the absorbance of sample extract and buffer system.

#### 3.3.3. Cell Proliferation Assay

In this research, the human umbilical vein endothelial cells (HUVECs) was provided by ScienCell, Co., LTD. [37,38]. Cell counting kit 8 (CCK-8) was used to determine the effect of COMF extract on the proliferation of HUVECs cells. COMF extract was diluted with PBS containing very small amount of DMSO. HUVECs cells were inoculated in 96-well plates and incubated with different concentrations of COMF extract (100, 50, 10, 5, 1, 0.5 and 0.1 μg/mL) for 24 h. According to the instructions of the kit, cell viability was measured by CCK-8 to indicate whether COMF extract itself had an effect on cell proliferation. In the control group, COMF extract was replaced with PBS. The absorbance at 450 nm was measured by enzyme calibration.

#### 3.3.4. Detection of Antioxidant Stress

HUVECs were damaged by H_2_O_2_ (200 μM) for oxidative stress detection. The dam-aged HUVECs were co-incubated with COMF extract at different concentrations (100, 10, 1 μg/mL) for 24 h. In the control group, the same amount of PBS was added instead of drugs. Model group was treated with H_2_O_2_ injury without COMF extract. Cell viability was determined by CCK-8 according to the kit detection method.

#### 3.3.5. Detection of Cellular NO

HUVECs induced by H_2_O_2_ (200 μM) were incubated with COMF extract of 100, 10, 1 μg/mL for 24 h. The blank group had no injury and no COMF. The model group was injured by H_2_O_2_, but no COMF extract was provided. According to the manufacturer’s protocol, co-culture supernatant measurement was taken and the NO Content Detection Kit was used to measure NO level in cell supernatant. The absorbance of the mixture was measured at 540 nm with enzyme calibration.

### 3.4. Prediction of Active Ingredients

Based on the remarkable α-glucosidase inhibitory activity of COMF, we decided to further explore the active components and mechanisms of COMF in the treatment of diabetes. Referring to the online database and Cytoscape software (version 3.9.0), we queried the targets of the UPLC-MS/MS representation components for clustering analysis and ranking of association degree with diabetic targets. Potential active ingredients were screened by their association with target sites. Comparing the literature [39], the active monomers for in-depth study were finally identified.

### 3.5. α-Glucosidase Inhibition Activity and Molecular Docking Analysis of Representative Flavonoids

The α-glucosidase inhibitory activity assay was used to detect the inhibitory activities of 7 representative flavonoids. A total of 20 μL COMF extract was replaced by flavonoids at different concentrations (0.001, 0.01, 0.1, 0.2, 0.5, 1.0, 2.0, 5.0 mg/mL). Other steps remained unchanged.

This study also explains the binding activity between the α-glucosidase and the active components. Molecular Docking Analysis. The 3D structures of key potential target proteins from the PDB database (http://www.wwpdb.org/ accessed on 5 November 2022) were downloaded, and saved in the .pdb format. The core component structures were obtained from the TCMSP database and also saved them in the .pdb format. The protein receptor molecules and core components were pre-treated by PyMOL software (version 2.2.0). The pre-processed potential targets and active ingredients were imported into the docking software AutoDock (version 1.5.7) for molecular docking, and the results were saved in the .pdbqt format. The resulting files were converted to the .pdb format by Open Babel GUI software (version 2.4.1), and molecular docking visualization analysis was performed by PyMOL software. The binding activity and possibility were judged according to the binding energy of ligand and receptor in the result file. It is generally believed that the lower the binding energy of ligand and receptor, the more stable the conformation of the binding. This study also explains the binding activity between the α-glucosidase (the PDB number of which is 2QMJ) and the active components.

### 3.6. Validation of the Molecular Mechanism

#### 3.6.1. Cell Proliferation Assay and Establishment of Cellular Glucose Damage Model

HUVECs cells were inoculated in 96-well plates and incubated with different concentrations of quercetin, kaempferol, isorhamnetin, luteolin and apigenin (all monomers were 10, 5.0 and 1.0 μM) for 24 h. According to the instructions of the kit, cell viability was measured by CCK-8 to indicate whether the components had an effect on cell proliferation. Cell viability was measured using CCK-8. In control group, the drug was replaced with PBS.

HUVECs in logarithmic growth phase were cultured in 96-well plates and synchronized for 24 h. After cell apposition, ECM medium containing 35 mM glucose was added for 24 h to construct a cell damage model, and then COMF extract (100 μg/mL) and active compounds (1.0 μM) were added and co-incubated for 24 h. Cells were used for RT-qPCR assay.

The Cont group was the control group, using normal culture of HUVECs cells without any cell damage or treatment manipulation. The Glu group was the model group, and the medium was co-cultured with 35 mM glucose without the addition of COMF extracts or compounds. The drug groups included the COMF extract group, positive drug group, quercetin group, kaempferol group, isorhamnetin group, luteolin group and apigenin group. While adding glucose to the cells, 100 μg/mL of COMF extract was given to the COMF extract group, 1.0 μM metformin was added to the positive drug group, and 1.0 μM of the corresponding compound was added to each compound group.

#### 3.6.2. Expression of AKT1, IL-6 and VEGFA mRNA by RT-qPCR

Cells were seeded in culture flasks and collected after drug administration. Total mRNA was extracted by Trizol reagent. Reverse transcription was performed according to the kit instructions. After the reaction was completed, the amplification reaction was carried out according to the instructions. The reaction conditions were 95 °C for 10 min, 95 °C for 15 s, and 60 °C for 1 min for 40 cycles. Each group was randomly divided into 3 samples, and each sample was divided into 3 duplicates for reverse transcription. The PCR amplification curve and melting curve were confirmed, and the cycle threshold (Ct value) of mRNA of each gene was recorded.

### 3.7. Statistical Analysis

Each experiment was repeated three times. Statistical analyses used IBM SPSS statis-tics Version 25.0 (IBM Corp., Armonk, New York, NY, USA). When the *p* value is less than or equal to 0.05, the difference of experimental data was considered statistically significant.

## 4. Discussion

*Cydonia oblonga* Mill. fruit is a traditional national medicine that dual-purpose for drug and food. In this study, the DPPH inhibition activity test and NO production test of oxidative damaged cells were used to verify that COMF had a good ability to resist oxidative stress. Antioxidant stress can improve the function of islet β-cells to a certain extent, thus contributing to the treatment of diabetes. COMF also showed significant α -glucosidase inhibitory activity. COMF extract at the concentration of 1-5 mg/mL could achieve an inhibition rate higher than 80%. α-Glucosidase is an important hydrolase involved in the process of glucose metabolism and its inhibition is one of the effective strategies to control T2DM.

Subsequently, we used UPLC-MS/MS to quickly identify the components in COMF, and combined it with bioinformatic analysis and RT-qPCR to discover the potential anti-DM active compounds and mechanisms. COMF total flavonoids and polysaccharides have a certain hypoglycemic effect [40]. However, the COMF composition identification needs to be supplemented, and the specific active ingredients that play a hypoglycemic role are still unclear. In this study, we used UPLC-MS to identify the components in COMF, a total of 65 compounds were identified by COMF, including flavonoids, polyphenols and organic acids and a few terpenoids. Flavonoids are a kind of important components with C6-C3-C6 skeleton, which exist widely in nature and have a broad spectrum of biological activities. Flavonoids can be divided into flavonoids, flavonols, flavane-3-alcohols, flavanones and other types according to their structure. Different types have different cleavage modes in mass spectrometry identification. Flavonols tend to break between positions 1–2 and 3–4 of the C-ring, whereas flavonols tend to break the bonds between positions 1–9 and 2–3. It is speculated that 4-OH has certain influence on the opening mode of flavone C-ring [41]. The polyphenols and organic acids in COMF mainly include caffeylquinic acid and cinnamic acid and their derivatives. The mass spectrum information of chlorogenic acid and its isomerism and isochlorogenic acid is similar, and the final results need to be determined by comparing the retention time of standard materials or literature records. Flavonoids, polyphenols and organic acids have extensive and diversified biological activities in nature, so it is of scientific significance to screen the activity and explore the mechanism of COMF components.

The potential active compounds of COMF were obtained by data mining and analysis. The results showed that flavonoids were very important. Flavonoids in traditional medicines have antioxidant activity, and play a role in protecting pancreatic β cells, regulating P38 MAPK signaling pathway, and protecting insulin secretion disorders [42,43,44]. Quercetin can reduce the apoptosis of TC6 insulin cells damaged by oxidative stress and trigger insulin secretion of glucose [45]. Apigenin regulates oxidative stress, apoptosis and inflammation through MAPK pathway, thereby improving streptozotocin induced diabetic nephropathy in rats [46]. Studies have shown that epicatechin’s antioxidant bioactivity regulates beta cell survival, proliferation and function [47]. In general, the main active substance of COMF against DM is flavonoids.

α-Glucosidase is an important hydrolase involved in the process of glucose metabolism and its inhibition is one of the effective strategies to regulate T2DM. COMF extract at the concentration of 1.0–5.0 mg/mL could achieve more than 80% inhibition rate, which showed that COMF had significant α-glucosidase inhibitory activity. The inhibition rate of seven representative flavonoids varies from 0.0090 to >5.0000 mg/mL. The binding energy values for molecular docking illustrate good binding simulations of the five flavonoid glycosides to key targets. However, rutin showed poor binding results, which are suspected to be related to the complex spatial structure of the glycoside that hinders the binding of the active site. Based on the docking results, the results of α-glucosidase activity inhibition were also better explained. Flavonoid aglycone showed stronger inhibitory activity against α-glucosidase than flavonoid aglycone rutin. However, flavane-3-ol epicatechin showed different inhibitory activity compared with docking results, and the reasons remain to be discussed.

The PI3K-AKT pathway is an important pathway in insulin signaling, and alterations in the protein amount and activity of key proteins in the pathway may lead to impairment of the pathway and decreased response to insulin, affecting glucose stability, which is one of the pathogenic mechanisms of DM. AKT1 has been shown to be expressed in insulin-sensitive tissues such as liver, skeletal muscle and adipose tissue. Activation of AKT enhances islet β cell survival [48]. It is noteworthy that the P13K-AKT pathway affects a variety of downstream effector molecules such as IL-6, NF-κB and IL-1β, which play an important role in the occurrence and development of diabetes through inflammatory immune response and insulin resistance [49]. IL-6 and IL-1β, as typical inflammatory targets, play an anti-oxidative stress role in the PI3K/AKT signaling pathway, AK/STAT signaling pathway and MAPK/ERK signaling pathway, indirectly affecting the activity and function of islet β -cells [50].

RT-qPCR results showed that COMF extract and quercetin, kaempferol, isorhamnetin, luteolin and apigenin were able to down-regulate IL-6 mRNA and up-regulate AKT1 and VEGFA mRNA expression in HUVECs cells injured by glucose with similar effects as the positive drugs. AKT1 and VEGFA are enriched targets in PI3K/AKT signaling pathway. Insulin receptor plays an important role in diabetes-related pathways, and diabetes mellitus usually shows abnormal number and affinity of insulin receptor [51]. AKT1 is one of three closely related serine/threonine protein kinases (AKT1, AKT2 and AKT3), called AKT kinase, which regulates the proliferation, survival, metabolism and angiogenesis of various cells. AKT depletion largely attenuates insulin signaling to glucose transporters and glycogen synthase kinases [52]. Vascular endothelial growth factor A (VEGFA) is a pro-angiogenic factor. COMF extract and active compounds could up-regulate VEGFA protein expression, promote VEGFA-mediated angiogenesis, and accelerate wound healing in diabetic patients. The important downstream effectors of PI3K/AKT signaling pathway are key nuclear factors in the initiation and regulation of inflammation, which can induce the expression of many inflammatory factors, such as IL-6, causing inflammatory response and aggravating the occurrence and development of diabetes [53]. AKT1, VEGFA and IL-6, as enriched targets or downstream key factors of the PI3K-AKT pathway, are relevant factors in the pathogenesis of diabetes. COMF and its active components quercetin, kaempferol, isorhamnetin, luteolin and apigenin could regulate AKT1, VEGFA and IL-6 mRNA levels and improve the dysregulation of PI3K-AKT pathway signaling to intervene the inflammation and trauma manifested during the development of diabetes.

## 5. Conclusions

At present, many anti-diabetes drugs have certain adverse reactions. It is urgent to find new anti-diabetes drugs with low toxicity and good efficacy. In this study, starting from the characterization of COMF components, through the study of its anti-diabetes activity in vitro and bioinformatics analysis, we screened the anti-diabetes active monomers and verified the molecular mechanism of the monomers. The results reveal that the active flavonoid monomer of COMF plays an anti-inflammatory and antioxidant role in the PI3K-AKT pathway, inhibiting the occurrence and development of diabetes. This study provides a clear idea for screening the activity of natural plant extracts, and also provides a basic study for exploring the mechanism of *Cydonia oblonga* Mill. against diabetes. It is hoped that in the near future, *Cydonia oblonga* Mill. and more natural products can become clinical anti-diabetes candidate drugs and play their multi active effects.

## Figures and Tables

**Table 1:** The data of components identified from COMF by ESI-Q-Exactive Orbitrap-MS/MS.

No.	tR/ Min	Precursor Ions	Detected/ m/z	Calculated/ m/z	Error/ppm	Molecular Formula	Fragment Ions/ m/z	Name	Class	Reference
1	0.88	[M + H]^+^	175.1187	175.1190	−1.612	C_6_H_14_N_4_O_2_	175.1187, 116.0707, 71.0691, 70.0658, 60.0564	Arginine	amino acid	[17]
2	1.12	[M + H]^+^	183.0860	183.0863	−1.719	C_6_H_14_O_6_	183.0860, 165.0546, 147.0648, 129.0546, 111.0442, 69.0341	Hexitol	other compound	[18]
3	1.15	[M + H]^+^	193.0704	193.0707	−1.215	C_7_H_12_O_6_	157.0494, 147.0649, 129.0546	Quinic acid	organic acid	[17]
4	1.43	[M + H]^+^	135.0286	135.0288	−1.184	C_4_H_6_O_5_	135.0286, 107.0493, 89.0390	Malic acid	organic acid	[19]
5	1.56	[M + H]^+^	118.0863	118.0863	0.803	C_5_H_11_NO_2_	118.0863, 72.0814, 59.0737, 58.0659	Valine	amino acid	[17]
6	1.75	[M − H]^−^	117.0179	117.0182	−2.950	C_4_H_6_O_4_	117.0179, 99.0072, 73.0279, 55.0173	Succinic Acid	organic acid	[17]
7	2.57	[M + H]^+^	175.0234	175.0237	−1.911	C_6_H_6_O_6_	175.0234, 139.0023, 67.0549	Shikimic acid	organic acid	[17]
8	2.58	[M − H]^−^	191.0190	191.0186	−3.904	C_6_H_8_O_7_	191.0190, 173.0085, 129.0180, 111.0073	Citric acid	organic acid	[20]
9	3.07	[M + H]^+^	132.1017	132.1019	−1.326	C_6_H_13_NO_2_	132.1017, 86.0969, 69.0705, 57.0343	Leucine	amino acid	[17]
10	3.85	[M + H]^+^	182.0808	182.0812	−1.866	C_9_H_11_NO_3_	182.0808, 165.0543, 147.0438, 136.0754, 123.0440	Tyrosine	amino acid	[19]
11	6.26	[M − H]^−^	169.0133	169.0131	0.948	C_7_H_6_O_5_	169.0133, 125.0230	Gallic acid	polyphenol	None ^1^
12	8.46	[M + H]^+^	331.0675	331.0660	1.420	C_13_H_16_O_10_	331.0675, 169.0132, 125.0230	Glucogallin	polyphenol	[21]
13	8.52	[M + H]^+^	166.0860	166.0863	−1.837	C_9_H_11_NO_2_	166.0860, 120.0808, 103.0545, 91.0546	Phenylalanine	amino acid	[17]
14	9.48	[M − H]^−^	153.0182	153.0182	−0.099	C_7_H_6_O_4_	153.0182, 109.0280	Protocatechuic acid	polyphenol	None ^1^
15	9.91	[M + H]^+^	579.1486	579.1497	−1.973	C_30_H_26_O_12_	427.1006, 409.0909, 289.0698, 127.0388	Procyanidin B1	flavonoid	[22]
16	10.50	[M + H]^+^	355.1014	355.1024	−2.587	C_16_H_18_O_9_	163.0386, 145.0281, 135.0438, 117.0335, 89.0389	neochlorogenic acid	polyphenol	None ^1^
17	10.67	[M − H]^−^	271.0829	271.0812	2.080	C_12_H_16_O_7_	271.0829, 109.0280, 101.0226, 71.0122	Arbutin	other compound	[23]
18	10.74	[M + H]^+^	579.1485	579.1497	−2.077	C_30_H_26_O_12_	427.1020, 409.0908, 289.0701, 127.0388	Procyanidin B2	flavonoid	[22]
19	10.79	[M − H]^−^	137.0232	137.0233	−0.953	C_7_H_6_O_3_	137.0232, 108.0201	3,4-Dihydroxybenzaldehyde	polyphenol	None ^1^
20	10.81	[M + H]^+^	139.0387	139.0390	−1.731	C_7_H_6_O_3_	139.0387, 111.0442, 95.0493, 65.0393	3-Hydroxybenzoic acid	organic acid	[24]
21	11.19	[M + H]^+^	355.1013	355.1024	−2.840	C_16_H_18_O_9_	163.0386, 145.0282, 135.0439, 117.0336, 89.0390	Chlorogenic acid	polyphenol	None ^1^
22	11.34	[M + H]^+^	291.0855	291.0863	−2.867	C_15_H_14_O_6_	291.0855, 273.0735, 161.0594, 147.0437, 139.0387, 123.0441	epicatechin	flavonoid	[22]
23	11.52	[M + H]^+^	169.0492	169.0495	−1.924	C_8_H_8_O_4_	169.0492, 151.0387, 123.0440, 93.0576	Vanillic acid	organic acid	[25]
24	11.65	[M + H]^+^	135.0544	153.0546	−1.768	C_8_H_8_O_3_	153.0544, 135.0803, 107.0848	4-Hydroxyphenylacetic acid	organic acid	[24]
25	11.69	[M + H]^+^	153.0544	153.0546	−1.572	C_8_H_8_O_3_	153.0544, 123.0440, 109.1014, 81.0704	Vanillin	other compound	[24]
26	11.88	[M + H]^+^	181.0492	181.0495	−1.907	C_9_H_8_O_4_	181.0492, 163.1115, 139.0751, 111.0443	Caffeic acid	organic acid	None ^1^
27	11.95	[M + H]^+^	355.1015	355.1024	−2.333	C_16_H_18_O_9_	355.1015, 193.0492, 163.0386	Cryptochlorogenic acid	polyphenol	[22]
28	12.17	[M + H]^+^	291.0857	291.0863	−2.249	C_15_H_14_O_6_	291.0857, 273.0747, 161.0595, 147.0438, 139.0387, 123.0441	catechin	flavonoid	None ^1^
29	12.87	[M + H]^+^	147.0438	147.0441	−1.673	C_9_H_6_O_2_	147.0438, 119.0492, 91.0546	Coumarin	organic acid	[26]
30	13.11	[M + H]^+^	369.1173	369.1180	−1.811	C_17_H_20_O_9_	351.1061, 177.0540, 145.0282	Methyl chlorogenate	polyphenol	[17]
31	13.25	[M + H]^+^	165.0544	165.0546	−1.458	C_9_H_8_O_3_	165.0544, 147.0438, 123.0441, 103.0545	2-Hydroxycinnamic acid	organic acid	[24]
32	13.29	[M + H]^+^	165.0544	165.0546	−1.276	C_9_H_8_O_3_	165.0544, 147.0438, 119.0492, 91.0546	4-Hydroxycinnamic acid	organic acid	[26]
33	13.37	[M + H]^+^	611.1595	611.1607	−1.818	C_27_H_30_O_16_	465.1052, 303.0492	rutin	flavonoid	None ^1^
34	13.52	[M + H]^+^	273.0751	273.0757	−2.490	C_15_H_12_O_5_	273.0751, 254.0443, 135.0801	Butein	other compound	[27]
35	13.64	[M + H]^+^	465.1020	465.1028	−1.704	C_21_H_20_O_12_	303.0492, 153.0180, 137.0232, 85.0289	Hyperoside	flavonoid	None ^1^
36	13.73	[M + H]^+^	465.1019	465.1028	−1.897	C_21_H_20_O_12_	303.0491, 153.0179, 137.0231, 85.0289	Isoquercitrin	flavonoid	[28]
37	13.76	[M + H]^+^	449.1070	449.1078	−1.799	C_21_H_20_O_11_	449.1070, 287.0542, 153.0179	Luteoloside	flavonoid	None ^1^
38	13.81	[M + H]^+^	195.0649	195.0652	−1.565	C_10_H_10_O_4_	177.0544, 163.0387, 149.0959, 145.0283, 117.0336	Ferulic acid	organic acid	None ^1^
39	14.04	[M + H]^+^	595.1646	595.1657	−2.010	C_27_H_30_O_15_	595.1657, 287.0543	Nicotiflorin	flavonoid	[22]
40	14.09	[M + H]^+^	149.0595	149.0597	−1.517	C_9_H_8_O_2_	149.0595, 131.0491, 103.0545	Cinnamic acid	organic acid	[25]
41	14.12	[M + H]^+^	517.1334	517.1341	−1.223	C_25_H_24_O_12_	517.1334, 499.1225, 163.0386. 145.0283, 135.0440, 117.0337, 89.0390	Isochlorogenic acid B	polyphenol	None ^1^
42	14.41	[M + H]^+^	449.1070	449.1078	−1.888	C_21_H_20_O_11_	449.1070, 287.0542	Astragalin	flavonoid	[29]
43	14.42	[M + H]^+^	517.1333	517.1341	−1.455	C_25_H_24_O_12_	517.1333, 499.1224, 163.0387. 145.0283, 135.0440, 117.0336, 89.0390	Isochlorogenic acid A	polyphenol	None ^1^
44	14.59	[M + H]^+^	611.1957	611.1970	−2.236	C_28_H_34_O_15_	611.1957, 303.0492	hesperidin	flavonoid	None ^1^
45	14.61	[M + H]^+^	449.1068	449.1078	−2.222	C_21_H_20_O_11_	449.1078, 431.1379, 303.0829	Quercitrin	flavonoid	[22]
46	14.69	[M + H]^+^	273.0750	273.0757	−2.820	C_15_H_12_O_5_	273.0750, 171.0286, 153.0179, 147.0437, 119.0492	naringenin	flavonoid	None ^1^
47	14.77	[M + H]^+^	517.1331	517.1341	−1.919	C_25_H_24_O_12_	517.1331, 499.1224, 163.0385. 145.0281, 135.0438, 117.0335, 89.0390	Isochlorogenic acid C	polyphenol	None ^1^
48	14.96	[M + H]^+^	189.1118	189.1121	−1.774	C_9_H_16_O_4_	189.1118, 171.1165, 161.1321, 147.0802	Azelaic acid	organic acid	[26]
49	15.46	[M + H]^+^	211.0961	211.0965	−1.968	C_11_H_14_O_4_	211.0961, 179.0699, 167.0700, 151.0387	Sinapyl alcohol	other compound	[24]
50	16.79	[M + H]^+^	287.0543	287.0550	−2.524	C_15_H_10_O_6_	287.0543, 269.0439, 153.0179, 135.0439	Luteolin	flavonoid	None ^1^
51	16.89	[M + H]^+^	303.0492	303.0499	−2.538	C_15_H_10_O_7_	303.0492, 285.0380, 257.0440, 229.0490	Quercetin	flavonoid	[30]
52	17.91	[M + H]^+^	271.0593	271.0601	−2.508	C_15_H_10_O_5_	271.0594, 243.0643, 153.0180, 119.0492	apigenin	flavonoid	None ^1^
53	18.09	[M + H]^+^	287.0542	287.0550	−2.942	C_15_H_10_O_6_	287.0542, 258.0515, 165.0543, 153.0181	Kaempferol	flavonoid	[30]
54	18.22	[M − H]^−^	271.0615	271.0601	1.082	C_15_H_12_O_5_	271.0615, 253.0506, 225.0557, 151.0024, 119.0484	pinobanksin	flavonoid	[31]
55	18.24	[M + H]^+^	181.0856	181.0859	−1.606	C_10_H_12_O_3_	181.0859, 163.1115, 145.1009, 123.0804	Coniferyl alcohol	other compound	[24]
56	18.27	[M + H]^+^	317.0649	317.1656	−2.205	C_16_H_12_O_7_	317.1016, 302.0779, 153.0543	Isorhamnetin	flavonoid	None ^1^
57	20.08	[M + H]^+^	375.1068	375.1074	−1.690	C_19_H_18_O_8_	375.1068, 360.1110	Casticin	flavonoid	[26]
58	20.42	[M + H]^+^	318.2994	318.3003	−2.798	C_18_H_39_NO_3_	318.2994, 300.2887, 282.2783	2-Aminooctadecane-1,3,4-triol	other compound	[32]
59	21.03	[M + H]^+^	287.0905	287.0914	−3.065	C_16_H_14_O_5_	287.0905, 272.0672, 244.0681	sakuranetin	flavonoid	[33]
60	21.46	[M + H]^+^	287.0905	287.0914	−2.961	C_16_H_14_O_5_	287.0905, 272.0669, 244.0703	Isosakuranetin	flavonoid	[33]
61	23.52	[M + H]^+^	473.3613	473.3625	−2.612	C_30_H_48_O_4_	473.3613, 455.3511, 409.3450	Maslinic acid	pentacyclic triterpenoids	[17]
62	23.75	[M + H]^+^	281.2467	281.2475	−2.975	C_18_H_32_O_2_	281.2467, 263.2369, 221.2256, 179.1425, 165.1273	linoleic acid	other compound	[17]
63	24.44	[M + H]^+^	277.2154	277.2162	−2.946	C_18_H_28_O_2_	277.2155, 93.0702, 79.0547	12-Phenyldodecanoic acid	organic acid	[34]
64	28.34	[M + H]^+^	457.3666	457.3676	−2.212	C_30_H_48_O_3_	457.3666, 439.3564, 411.3608, 249.1844	oleanolic acid	pentacyclic triterpenoids	[17]
65	28.88	[M + H]^+^	457.3664	457.3676	−2.759	C_30_H_48_O_3_	457.3664, 439.3557, 411.3609, 393.3507	Ursolic Acid	pentacyclic triterpenoids	None ^1^

^1^ None stands for identification of cracking rule using reference standard.

**Table 2:** The active compounds and their parameter information. ASPL, BC, CC and degree in the table correspond to average shortest path length, betweenness centrality and closeness centrality and degree.

Compound	ASPL	BC	CC	Degree
Quercetin	1.69090909	0.20754625	0.59139785	98
Apigenin	2.39393939	0.03164871	0.41772152	39
Ursolic Acid	2.43030303	0.03084614	0.41147132	36
Kaempferol	2.45454545	0.02240119	0.40740741	35
Luteolin	2.45454545	0.02116046	0.40740741	34
Epicatechin	2.61212121	0.01209597	0.38283063	21
Isorhamnetin	2.64848485	0.00989390	0.37757437	18
Rutin	2.67272727	0.00675207	0.37414966	16
Casticin	2.78181818	0.00134943	0.35947712	7
Quercitrin	2.78181818	0.00109912	0.35947712	7
Astragalin	2.78181818	0.00155056	0.35947712	7
Oleanolic acid	2.78181818	0.00128503	0.35947712	7
Hesperidin	2.79393939	0.00069670	0.35791757	6
Catechin	2.79393939	0.00108638	0.35791757	6
Procyanidin B2	2.80606061	0.00118610	0.35637149	5
Hyperoside	2.80606061	0.00031936	0.35637149	5
Luteoloside	2.81818182	0.00021088	0.35483871	4
Butein	2.83030303	0.00006069	0.35331906	3
Procyanidin B1	2.83030303	0.00006069	0.35331906	3
Cryptochlorogenic acid	2.84242424	0.00001026	0.35181237	2
Nicotiflorin	2.84242424	0.00013911	0.35181237	2
Chlorogenic acid	2.84242424	0.00001026	0.35181237	2

**Table 3:** IC50 values and molecular docking binding energies of 7 representative flavonoids.

Name	IC50 (mg/mL)	Binding Energy (KJ/mol)
Acarbose	<0.0010	−44.81
Quercetin	0.0090	−30.88
Kaempferol	0.0646	−29.12
Isorhamnetin	0.1588	−29.33
Luteolin	0.1904	−32.97
Apigenin	0.2508	−32.17
Rutin	1.4790	−13.01

## Data Availability

Data is contained within the article.
